# Characterization of diabetic neuropathy progression in a mouse model of type 2 diabetes mellitus

**DOI:** 10.1242/bio.036830

**Published:** 2018-08-06

**Authors:** Cristian De Gregorio, David Contador, Mario Campero, Marcelo Ezquer, Fernando Ezquer

**Affiliations:** 1 Center for Regenerative Medicine, Facultad de Medicina, Clínica Alemana- Universidad del Desarrollo, Santiago 7710162, Chile; 2 Department of Neurology & Neurosurgery, Hospital José Joaquín Aguirre, Universidad de Chile, Santiago 7710162, Chile; 3 Departamento de Neurología, Clínica Las Condes, Santiago 7710162, Chile

**Keywords:** Diabetic neuropathy, Type 2 diabetes mellitus, Animal model, Peripheral nerves, Axonal degeneration

## Abstract

Diabetes mellitus (DM) is one of most common chronic diseases with an increasing incidence in most countries. Diabetic neuropathy (DN) is one of the earliest and main complications of diabetic patients, which is characterized by progressive, distal-to-proximal degeneration of peripheral nerves. The cellular and molecular mechanisms that trigger DN are highly complex, heterogeneous and not completely known. Animal models have constituted a valuable tool for understanding diabetes pathophysiology; however, the temporal course of DN progression in animal models of type 2 diabetes (T2DM) is not completely understood. In this work, we characterized the onset and progression of DN in BKS diabetic (*db/db*) mice, including the main functional and histological features observed in the human disease. We demonstrated that diabetic animals display progressive sensory loss and electrophysiological impairments in the early-to-mid phases of the disease. Furthermore, we detected an early decrease in intraepidermal nerve fiber (IENF) density in 18-week-old diabetic mice, which is highly associated with sensory loss and constitutes a reliable marker of DN. Other common histological parameters of DN – like Schwann cells apoptosis and infiltration of CD3^+^ cells in the sciatic nerve – were altered in mid-to-late phases of the disease. Our results support the general consensus that DN evolves from initial functional to late structural changes. This work aimed to characterize the progression of DN in a reliable animal model sharing the main human disease features, which is necessary to assess new therapies for this complex disease. Finally, we also aimed to identify an effective temporal window where these potential treatments could be successfully applied.

## Abstract (precis)

**Summary:** We characterized the main functional and structural diabetic neuropathy features during early-to-late phases of type 2 diabetes mellitus. This study aimed to identify a therapeutic window for new treatments.

## INTRODUCTION

Diabetes mellitus (DM) is one of most common chronic diseases, reaching epidemic level with approximately 425 million patients worldwide (International Diabetes Federation, 2017). T2DM is the most common form of DM, which accounts for at least 90% of all cases, and its incidence is increasing in most countries.

One of the earliest and main complications in diabetic patients is diabetic neuropathy (DN), occurring in approximately 60% of DM cases (type 1 and 2) (Vincent and Feldman, 2004). DN is characterized by progressive, distal-to-proximal degeneration of peripheral nerves, affecting sensory, motor and autonomic fibers, which results in sensory loss, muscle weakness and pain (Aring et al., 2005). According to NIH's Diabetic Complications Consortium (DiaComp), DN diagnosis requires: (i) evaluation of sensory loss, (ii) nerve conduction velocity test, and (iii) identification of fiber anatomical defects, as reduction of intraepidermal nerve fiber (IENF) density or fiber myelination studies.

Clinical trials have mainly focused on achieving an effective control of blood glucose levels, but there are no therapies to prevent or cure DN. Most of the DN treatments are ineffective and have side effects (Singh et al., 2014), deeply affecting patients' quality of life. Therefore, it is highly relevant to identify the main functional and structural events occurring during DN progression to develop better therapies.

The cellular and molecular mechanisms that trigger DN are complex and still remain highly unknown (Feldman et al., 2017). However, there is strong evidence indicating that hyperglycemia produces metabolic and physiological abnormalities that affect the nerve microenvironment, including an increase of reactive oxygen species (ROS), a sustained pro-inflammatory status and a decrease in the local blood flow (Figueroa-Romero et al., 2008; Vincent et al., 2011). Animal models have constituted a valuable tool for understanding diabetes pathophysiology. There are an important number of diabetic animal models that have been successfully validated, including genetic, diet-induced and chemical-induced models (Sullivan et al., 2007; Islam, 2013; O'Brien et al., 2014). These animal models have evidenced functional, biochemical and morphological changes in the retina, kidney and peripheral nerves, similar to those observed in humans (Sullivan et al., 2007; Nowicki et al., 2012; Islam, 2013; O'Brien et al., 2014). However, the onset and progression of DN in these rodent models are not completely understood.

A powerful mouse model of DN should exhibit the key features present in human disease, including sensory loss, electrophysiological defects and histological evidence of decreased IENFs. Sullivan and collaborators (Sullivan et al., 2007) showed that BKS *db/db* diabetic mouse line was one of the most robust models for DN. This animal model has a point mutation in the leptin receptor-coding gene (Ob-R), which affects specifically the long form receptor (Ob-Rb), but not the short isoforms (Chen et al., 1996; Madiehe et al., 2003). This mutation is sufficient to induce hyperphagia in *db/db* animals, leading to a rapid and sustained increase in body weight, dyslipidemia, insulin resistance and induction of severe hyperglycemia after the fourth week of life. Thus, *db/db* mice exhibited the main features observed in the T2DM human pathology (Wang et al., 2014; Campero et al., 2015).

The main goal of this study was to characterize the onset and progression of DN in this T2DM model. To accomplish this, we analyzed sensory loss, electrophysiological parameters and histology of diabetic mice in early, mid and late phases of the disease. This work constitutes a significant starting point for the development of new therapeutic trials for DN, and to identify an effective temporal window where these potential treatments could be successfully applied.

## RESULTS

### Glycemia, body weight and biochemical parameters through diabetes development

Previously, it has been described that *db/db* mice developed obesity from four weeks and hyperglycemia from four to eight weeks of age (Sullivan et al., 2007; Wang et al., 2014; O'Brien et al., 2014). Here, we measured non-fasting glucose levels and body weight from week 4–32 to evaluate the onset and progression of the disease. The body weight of *db/db* mice was similar to their control littermates until four weeks of age, but they then presented a strong weight gain until 26 weeks of age, where it plateaued (Fig. S1A). Likewise, *db/db* mice showed normal glycemia levels at four weeks of age, but this value was tripled by week 10 and quadrupled by week 32 (Fig. S1B). Diabetic mice presented a significant increase in plasmatic triglyceride levels (53.4±12.3 mg/dl in *db/+* mice versus 261.6±86.1 in *db/db* mice, *P*<0.001 by Student’s *t*-test) and glycated hemoglobin levels (3.8±0.2% in *db/+* mice versus 13.8±0.2% in *db/db* mice, *P*<0.001 by Student’s *t*-test) at 32 weeks of age compared to normal mice. Thus*, db/db* mice could be considered as a suitable T2DM model from 10 weeks of age.

### Behavioral and physiological testing

**Fig. 1.**
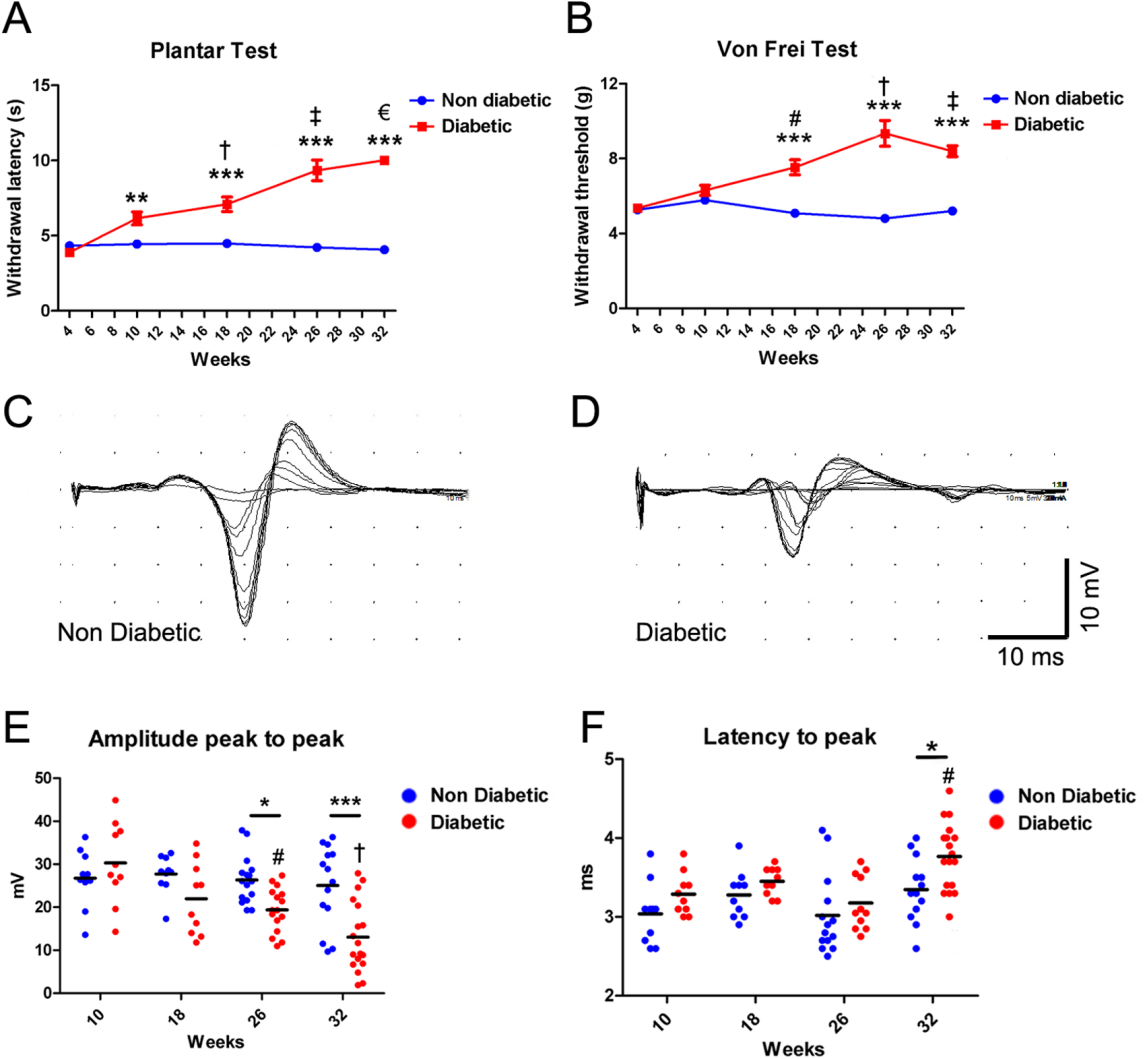
**Sensory loss and electrophysiological impairments in *db/db* mice.** (A) Hargreaves Plantar Test showing the withdrawal latency to a thermal stimulus in diabetic and non-diabetic mice between 4–32 weeks of age. Asterisks indicate significant differences between diabetic mice and their age-matched control littermates (*n*≥10, two-way ANOVA with Bonferroni post-test). Other symbols represent significant differences between diabetic mice (#, 10-week-old versus 4­-week-old mice; †, 18-week-old versus 4-week-old mice; ‡, 26-week-old versus 4-, 10- and 18-week-old mice; €, 32-week-old versus 4-, 10- and 18-week-old mice). (B) Von Frey Test showing the withdrawal threshold to a mechanical stimulus in diabetic and non-diabetic mice between 4–32 weeks of age. Asterisks indicate significant differences between diabetic mice and their age-matched control littermates (*n*≥10, two-way ANOVA with Bonferroni post-test). Other symbols represent significant differences between diabetic mice (#, 18-week-old versus 4-week-old mice; †, 26-week-old versus 4- and 10-week-old mice; ‡, 32-week-old versus 4- and 10-week-old mice). (C,D) Representative examples of CMAP recordings in sciatic nerves from non-diabetic (C) and diabetic mice (D) at 32 weeks of age. (E) Quantification of CMAP amplitude (from peak to peak) in diabetic and non-diabetic mice of 10–32 weeks of age. Data are presented as mean±s.e.m. Asterisks indicate significant differences between diabetic mice and their age-matched control littermates (*n*≥10, two-way ANOVA with Bonferroni post-test). Other symbols represent significant differences between diabetic mice (#, 26-week-old versus 10-week-old mice; †, 32-week-old versus 10- and 18-week-old mice). (F) Quantification of latency to peak in diabetic and non-diabetic mice of 10–32 weeks of age. Data are presented as mean±s.e.m. (*n*≥10, two-way ANOVA with Bonferroni post-test). # indicates significant differences between diabetic mice (32-week-old versus 10- and 26-week-old mice). *P<0.05, **P<0.01, ***P<0.001.

To analyze the evolution of DN symptoms during diabetes progression, we evaluated the main physiological features present in the human disease, including sensory loss and nerve electrophysiological impairments. First, we quantified the withdrawal response to thermal and mechanical stimulation in mice from 4–32 weeks of age by plantar and Von Frey assays, respectively. Using the plantar test, we found that diabetic mice displayed decreased heat sensitivity from 10 weeks of age, and that these responses worsened by 18 and 26 weeks of age, where the impairment reached a plateau (Fig. 1A). On the other hand, *db/db* mice showed no significant differences in mechanical response to Von Frey filament at 4 and 10 weeks of age, but displayed an increased threshold from 18 weeks of age compared with aged-matched normal mice (Fig. 1B). These results indicate that diabetic mice present normal or mild sensorial response impairments at 10 weeks of age, which worsened in a progressive manner until 26 weeks of age (one-way ANOVA analysis; Fig. 1A and B). We did not detect significant differences in these measurements between 26 and 32 weeks in diabetic mice, suggesting that these impairments leveled off.

Next, to study the electrophysiological changes in peripheral nerves we measured the compound motor action potential (CMAP) in the hind paw by stimulating the sciatic nerves of diabetic and control mice at 10, 18, 26 and 32 weeks of age. Electrophysiological recordings derived from diabetic mice nerves showed decreased CMAP amplitude from 26 weeks of age compared with control littermates, and this response worsened until 32 weeks of age (Fig. 1D and E). Furthermore, diabetic mice CMAP recordings presented an increased mean peak latency at 32 weeks of age, a parameter that is directly related to nerve conduction velocity (Fig. 1D and F).

Taken together, these results indicate than *db/db* mice present electrophysiological impairments that worsened over time, according to diabetes progression. Based in the severity of these physiological alterations, we defined four phases of DN to analyze the progression of other neuropathy-associated histological criteria: a very early (10 weeks), an early (18 weeks), a mid (26 weeks) and a late (32 weeks) phase of the disease.

### Intraepidermal nerve fiber density analysis

**Fig. 2.**
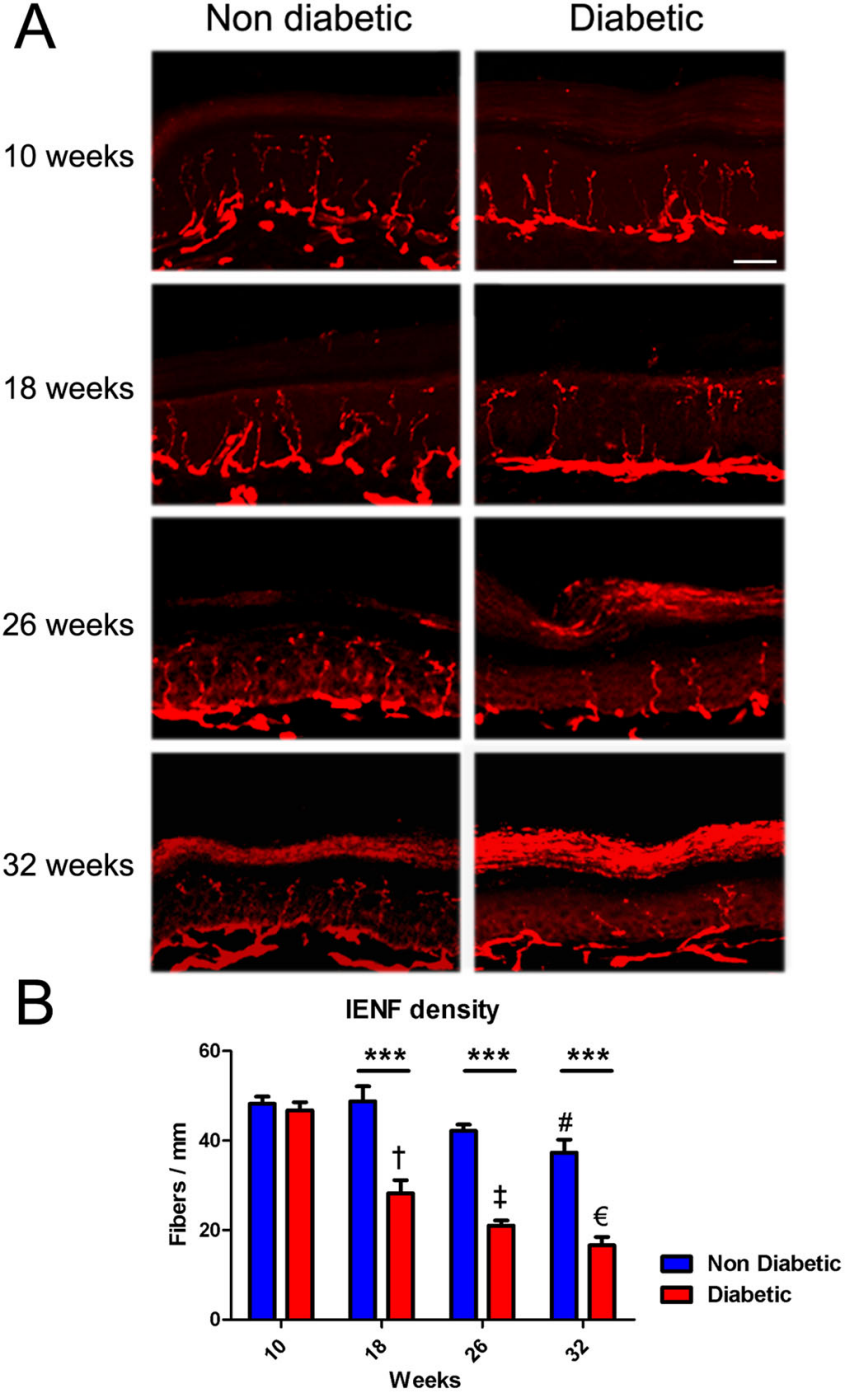
**Plantar skin innervations loss in *db/db* mice.** (A) Representative confocal images of IENF (stained with PGP 9.5) from plantar skin samples derived from diabetic and non-diabetic mice (10, 18, 26 and 32 weeks of age). (B) Quantification of the number of fibers crossing from dermis to epidermis per mm of skin length. Data are presented as mean±s.e.m. Asterisks indicate significant differences between diabetic mice and their age-matched control littermates (*n*=6, two-way ANOVA with Bonferroni post-test). Other symbols represent significant differences between non-diabetic mice (# 32-week-old versus 10- and 18-week-old mice) and between diabetic mice (†, 18-week-old versus 10-week-old mice; ‡, 26-week-old versus 10-week-old mice; €, 32-week-old versus 10- and 18-week-old mice). Scale bar: 50 µm. ***P>0.001.

IENF are directly associated with functional innervations of the skin. IENF density decline constitutes one of main histological features in human DN, and has been previously assessed in diverse diabetes models. To analyze whether fiber reduction is related to T2DM progression, we obtained hind limb plantar skin samples from 10-, 18-, 26- and 32-week-old diabetic mice and their control littermates. To quantify the IENF density, we carried out an immunodetection of the PGP 9.5 antigen in cryosectioned skin samples. We quantified the number of fibers crossing from the dermis to epidermis per linear mm of skin. Our results indicates that diabetic mice displayed a significant decrease in IENF density from 18 weeks of age compared to its littermates, and these differences worsened in 26- and 32-week-old diabetic mice, evidencing progressive impairments that correlate with disease evolution (Fig. 2). Interestingly, we also detected a mild decrease in IENF density in aged non-diabetic (*db/+*) mice, which could be related to a normal aging process (Fig. 2B, one-way ANOVA analysis).

### Apoptosis of Schwann cells in sciatic nerves

**Fig. 3.**
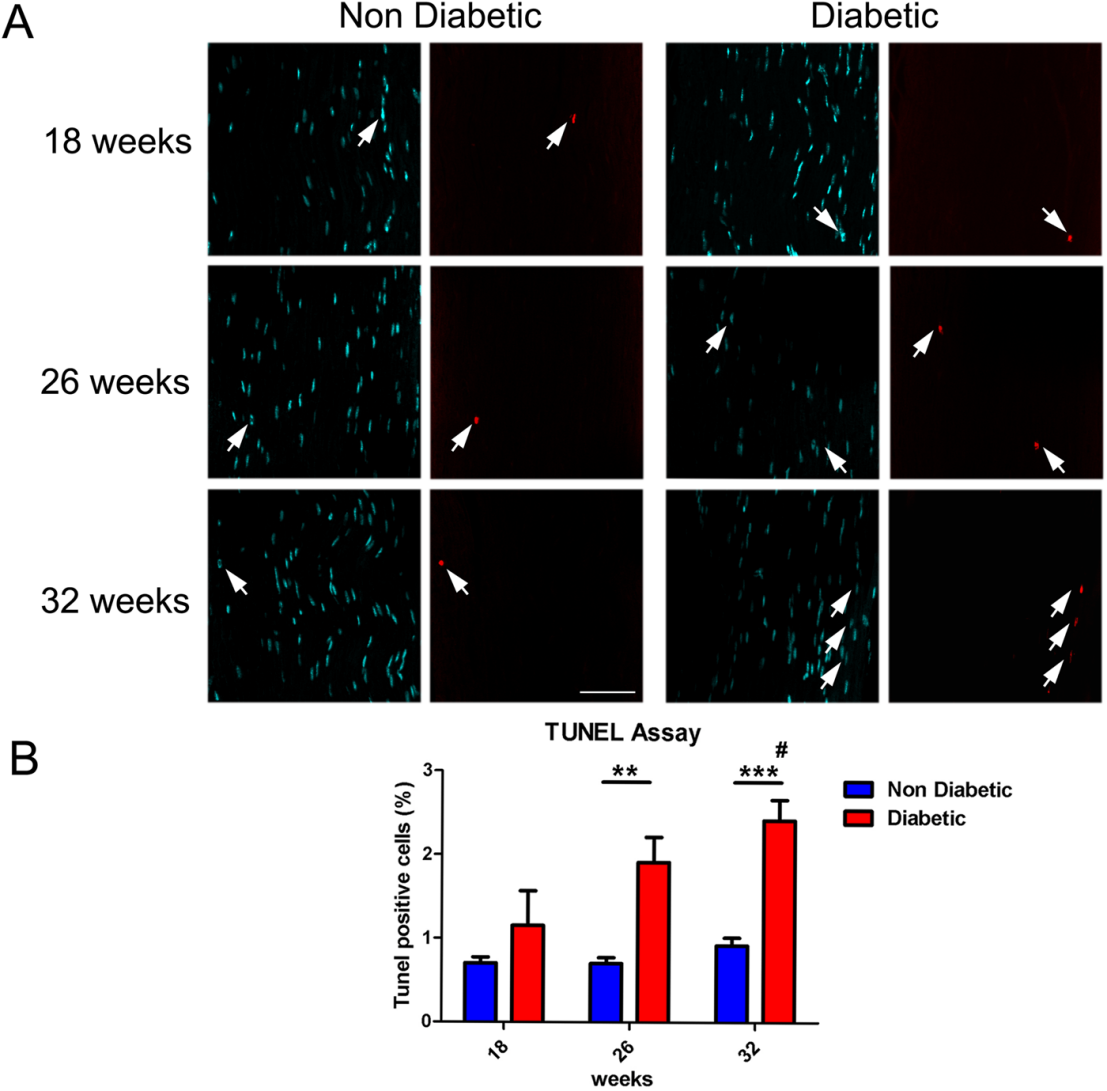
**Schwann cell apoptosis in sciatic nerves of *db/db* mice.** (A) Representative confocal images of apoptotic Schwann cells labeled with the TUNEL staining in sciatic nerves from diabetic and non-diabetic mice (18, 26 and 32 weeks of age). Nuclei were counterstained with DAPI. White arrows indicate TUNEL^+^ cells. (B) Quantification of TUNEL^+^ cells in sciatic nerves. Data are presented as mean TUNEL^+^ cells±s.e.m. (*n*=6, two-way ANOVA with Bonferroni post-test). Asterisks represent significant differences between diabetic mice and their age-matched control littermates. # represents significant differences between diabetic mice (32-week-old versus 18-week-old mice). Scale bar: 50 µm. **P<0.01, ***P<0.001.

Previously, it has been reported that due to the high mitochondrial density in Schwann cells, these cells are affected early by hyperglycemia and an oxidative environment in the diabetic condition, resulting in apoptosis (Wu et al., 2013; Zhu et al., 2018). To analyze whether Schwann cells are susceptible to this noxious microenvironment during diabetes progression, we carried out a TUNEL assay in sciatic nerves, derived from diabetic and non-diabetic mice, and quantified the percentage of TUNEL^+^ cells. No significant differences were found in 18-week-old animals, but we detected a significant increase in TUNEL^+^ cells at 26 weeks with a further increase in 32-week-old animals compared with aged-matched normal littermates (Fig. 3A and B). These results suggest that Schwann cells are particularly vulnerable to apoptosis during the mid-to-late phases of the disease. Whether this result involves physiological consequences or whether it constitutes a secondary consequence derived from other pathways remains to be explored. As Schwann cells are important regulators of nerve function, these could be assayed as an interesting target to avoid or slow the peripheral nerve degeneration in DN.

### Infiltration of T cells in the sciatic nerve

**Fig. 4.**
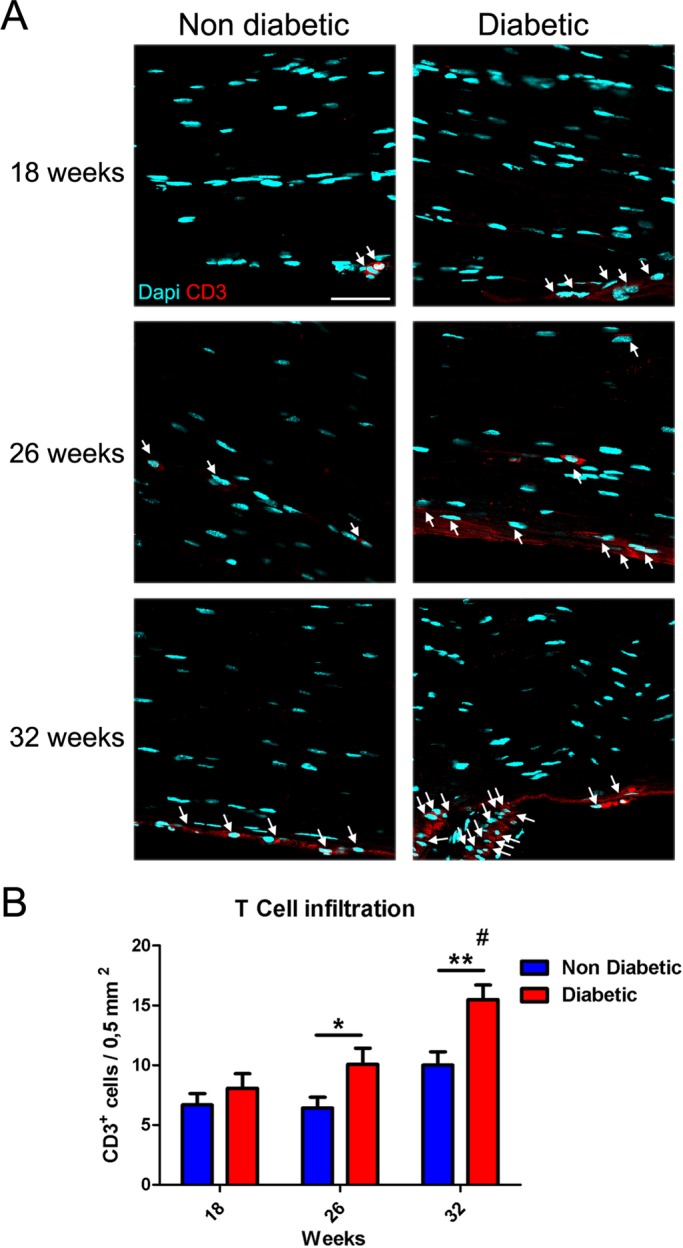
**T cell infiltration in sciatic nerves of *db/db* mice.** (A) Representative confocal images of cross sections of sciatic nerves derived from diabetic and non-diabetic mice (18, 26 and 32 weeks of age). Samples were stained against T lymphocyte marker CD3, and nuclei were counterstained with DAPI. T lymphocytes are denoted by white arrows. (B) Quantification of CD3^+^ cells in sciatic nerves. Data are presented as mean CD3^+^ cells per area±s.e.m. (*n*=6, two-way ANOVA with Bonferroni post-test). Asterisks represent significant differences between diabetic and their control littermates. # represents significant differences between diabetic mice (32-week-old versus 18- and 26-week-old mice). Scale bar: 50 µm. *P<0.05, **P<0.01.

To study the response of T lymphocytes and the inflammatory state of peripheral nerves through diabetes onset and progression, we analyzed the infiltration of CD3 positive cells in the sciatic nerve. We surgically obtained the sciatic nerves from diabetic and non-diabetic mice at 18, 26 and 32 weeks of age, and carried out an immunofluorescence analysis against the CD3 antigen. A significant increase of T lymphocytes was observed from week 26 exclusively in diabetic mice, and this difference increased in 32-week-old diabetic mice compared with their control littermates (Fig. 4A and B, one-way ANOVA analysis). These results indicate that T lymphocytes are slowly accumulated in the sciatic nerve during diabetes progression, and suggest that diabetic mice present an increased local inflammation level in their peripheral nerves. It remains to be determined if this local inflammation state is a major contribution to DN clinical symptoms.

### Sciatic nerve microvessels

**Fig. 5.**
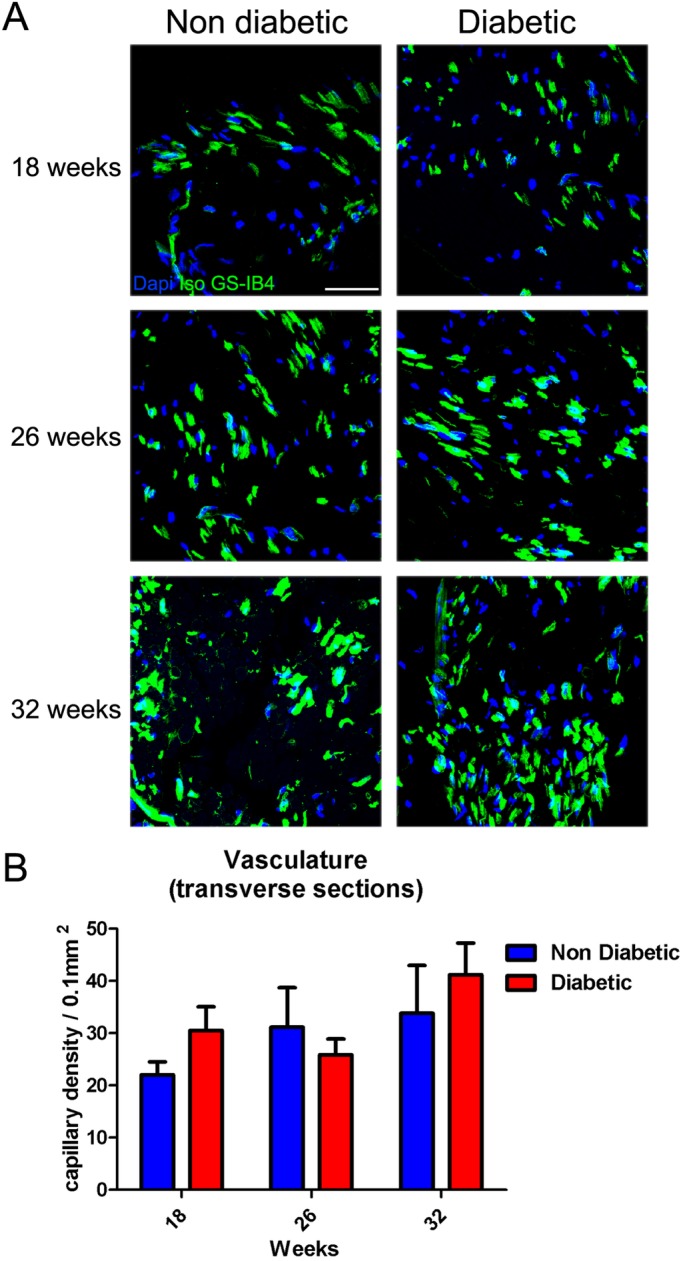
**Transverse *vasa nervorum* analysis in sciatic nerves of *db/db* mice.** (A) Representative confocal images of cross sections of sciatic nerves derived from diabetic and non-diabetic mice (18, 26 and 32 weeks of age). Samples were stained with Isolectin GS-IB4 and nuclei were counterstained with Hoechst. (B) Quantification of capillary density in sciatic nerves. Data are presented as mean capillary density per area±s.e.m. (*n*=6, two-way ANOVA with Bonferroni post-test). Scale bar: 50 µm.

**Fig. 6.**
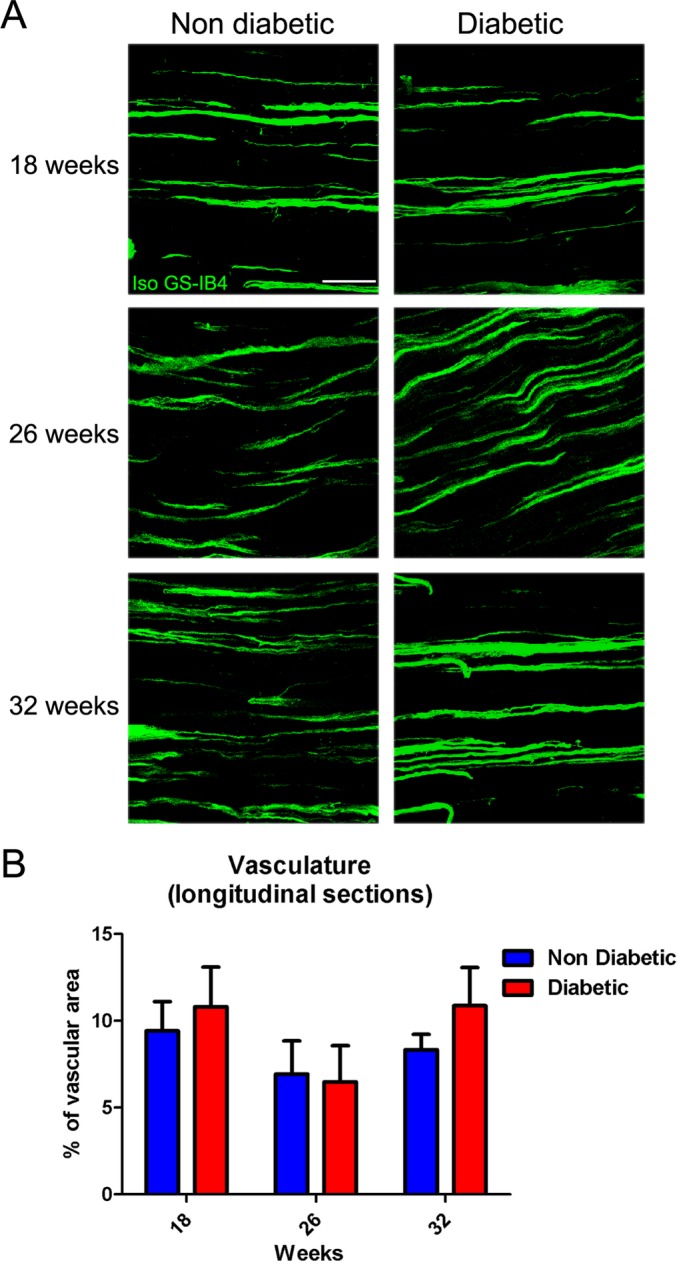
**Longitudinal *vasa nervorum* analysis in sciatic nerves of *db/db* mice.** (A) Representative confocal images of longitudinal sections of sciatic nerves derived from diabetic and non-diabetic mice (18, 26 and 32 weeks of age). Samples were stained with Isolectin GS-IB4. (B) Quantification of vascular area in sciatic nerves. Data are presented as mean area±s.e.m. (*n*=6, two-way ANOVA with Bonferroni post-test). Scale bar: 50 µm.

To explore the histological changes in blood vessels of peripheral nerves during diabetes progression, we surgically obtained the sciatic nerves from diabetic and non-diabetic mice at 18, 26 and 32 weeks of age. The number of microvessels was determined from nerve cross sections stained with fluorescent isolectin GS-IB4. No significant differences were observed in the total number of vessels in diabetic mice compared with their control littermates at any analyzed time (Fig. 5A and B). We also analyzed the nerve vascularization in longitudinal sections of the entire sciatic nerve. However, no differences were detected in the vessels area in any of the evaluated conditions (Fig. 6A and B).

### Sciatic nerve fiber morphology

**Fig. 7.**
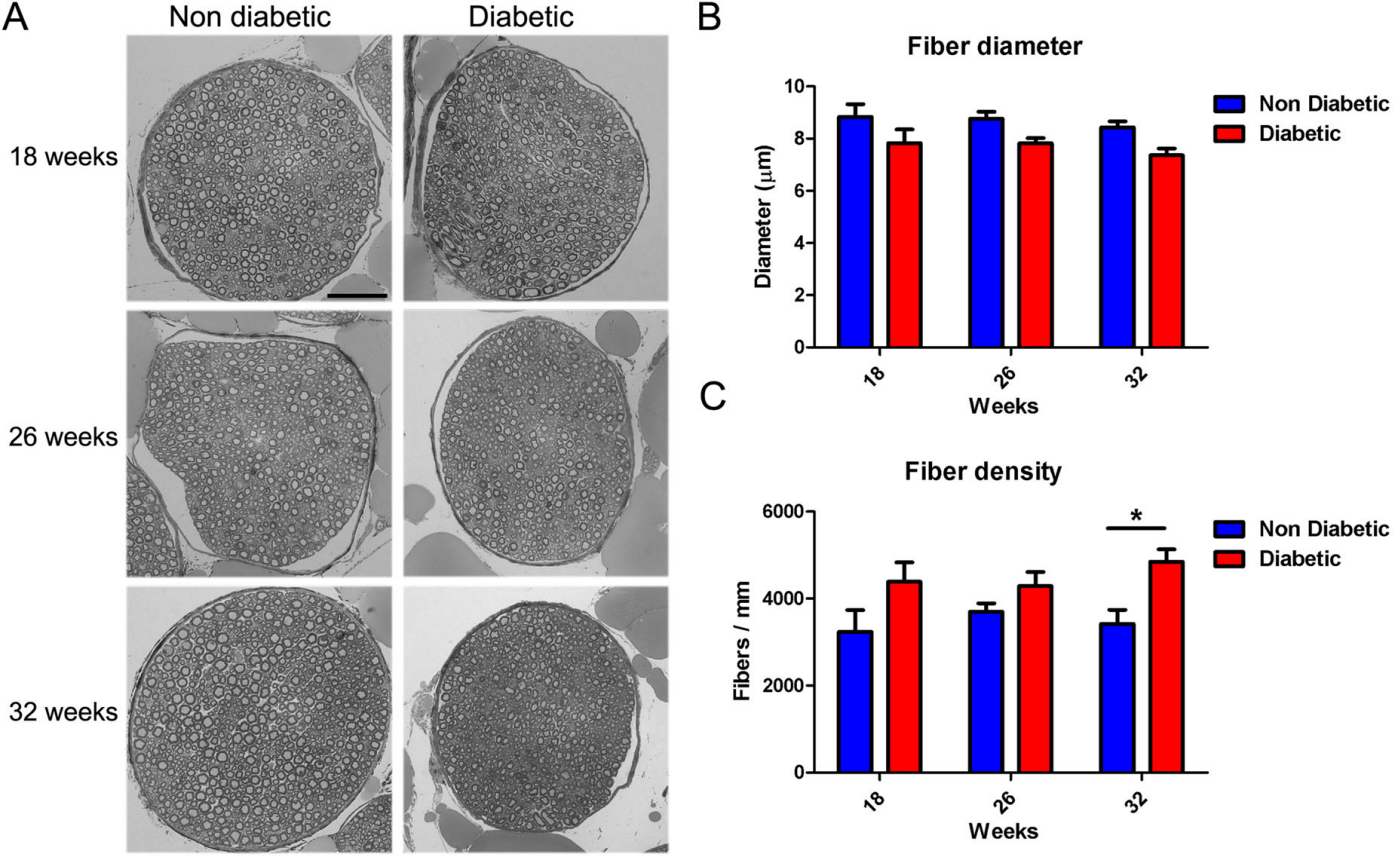
**Fiber changes analysis in sciatic nerves of *db/db* mice.** (A) Representative bright-field images of semithin sections of sciatic nerves derived from diabetic and non-diabetic mice (18, 26 and 32 weeks of age). Myelinated fibers were stained with Toluidine Blue. (B) Quantification of diameter of nerve fibers in sciatic nerve. Data are presented as mean diameter±s.e.m. (*n*=6, two-way ANOVA with Bonferroni post-test). (C) Quantification of fiber density in the sciatic nerve. Data are presented as mean fiber number per area±s.e.m. (*n*=6, two-way ANOVA with Bonferroni post-test). Scale bar: 50 µm. *P<0.05.

Previous reports have shown that *db/db* diabetic mice displayed alterations in sciatic nerve fiber morphology (Sima and Robertson, 1978, 1979; Carson et al., 1980; Nowicki et al., 2012; Campero et al., 2015). To explore the temporal curse of these changes, we obtained semithin sections from sciatic nerves from the early-to-late phase of the disease. Sciatic nerves derived from diabetic mice did not shown significant changes in the main diameter of the fibers compared with control littermates at any analyzed time (Fig. 7A and B); however, we detected an increased number of fibers (expressed as fiber density) in diabetic mice at 32 weeks of age compared to age-matched control mice (Fig. 7A and C). When we analyzed the size distribution of these fibers, we observed a significant decrease in large fibers (>13 µm diameter fibers) in diabetic mice at 18, 26 and 32 weeks of age compared with normal mice, suggesting that large fibers are more susceptible to diabetic condition (Fig. S2). Furthermore, 32-week-old diabetic mice displayed an increase in small fibers compared with their control littermates (< 4 µm diameter fibers, Fig. S2C). These results indicate that peripheral nerves of diabetic mice suffered mild but progressive morphological changes that may contribute to nerve electrophysiological impairments.

## DISCUSSION

In humans, DN progression has been described as a clinical complication that evolves from functional impairments in the early phases of the disease to structural/histological changes at advanced states (Boucek, 2006). This temporal course has not been easy to replicate in animal models (including genetic, chemical-induced and diet-induced) because a vast heterogeneity in DN associated-parameters exists (O'Brien et al., 2014). Animal models constitute key elements to understand the cellular and physiological principles of a disease, but also to model potential new therapeutic trials. BKS *db/db* mice are probably the most used model of T2DM, because these animals develop severe hyperglycemia and display advanced stages of disease relatively earlier compared with other models (Sullivan et al., 2007; Wattiez et al., 2012; Islam, 2013). Other advantages of this model are that they do not require specific diets, invasive procedures or pharmacological treatments that could induce unwanted side effects. A considerable disadvantage of *db/db* mice is the increased leptin plasmatic level, which has been related to other physiological processes like immune response modulation, energy homeostasis, reproduction, skeletal growth, neuroprotection and oncogenesis (Veniant and LeBel, 2003; Wang et al., 2014). Furthermore, despite leptin signaling blocking having been related to diabetes development in rodents, it seems to be less critical in humans (Veniant and LeBel, 2003).

In this work, we evaluated the main physiological and structural features of DN from the early-to-late phases of the disease, which could enable understanding of the progression of the most relevant clinical signs across the whole disease. At a functional level, diabetic mice displayed reduced responses to thermal and mechanical stimulus in early phase of disease (10–18 weeks of age), and this sensitivity loss worsened during later phases of the disease. Although allodynia has been described in some rodent models of DN during early phases of diabetes (Calcutt et al., 1996; Cheng et al., 2009; Fuchs et al., 2010; Shi et al., 2013; O'Brien et al., 2014; Brini et al., 2017), we did not observe signs of exacerbated response nor pain to mechanical and thermal stimulus in *db/db* mice. Even in human patients, mechanical sensitivity can oscillate from no sensation to severe mechanical allodynia. Thus, experimental evidence suggests that tactile and thermal sensitivity during early phase of diabetes is determined by complex regulation; meanwhile the sensitivity loss during the advanced phases of the disease has been well demonstrated in patients and animal models.

Cutaneous sensory neurons include unmyelinated C-fibers and myelinated Aδ-fibers and Aβ-fibers (Cain et al., 2001; Abraira and Ginty, 2013). Thermal stimuli and mechanical sensitivity are determined mainly by C-type small-fibers and myelinated Aδ-fibers (Abraira and Ginty, 2013). Interestingly, in human patients a strong correlation between thermal sensory loss and reduced IENF density has been described (Pan et al., 2001; Shun et al., 2004; Pittenger et al., 2004). Furthermore, IENF density was negatively correlated with diabetes duration in patients (Shun et al., 2004), which reinforces our results that IENF is an early and progressive DN marker. Thus, IENF analysis has emerged as a reliable diagnostic tool to evaluate DN progression.

Additionally, our electrophysiological studies showed a progressive reduction in CMAP amplitude and extended motor latencies in mid-to-advanced phases of diabetes (26- and 32-week-old mice). Although some authors have previously reported a decreased motor nerve conduction velocity from 16 weeks in the same diabetic line (Sima and Robertson, 1978; Norido et al.; 1984), others have reported alterations only after 24 weeks of hyperglycemia (28-week-old mice; Sullivan et al., 2007) or in 25-week-old mice (Moore et al., 1980). Additionally, Whiteley and Tomlison (1985) did not find significant differences in nerve conduction velocity in 20-week-old mice, suggesting variations in the onset of physiological nerve impairments in *db/db* mice in diverse laboratories. These differences could be attributed mainly to diet composition and sex (indeed, males have a more pronounced diabetic phenotype than females). Nevertheless, our results confirmed that motor nerve conduction impairments are present in mid-to-late stages of diabetes, in an age-dependent manner, as happens in the human disease.

The differences in electrophysiological parameters may be related to the morphological changes observed in sciatic nerve fibers. Diabetic mice displayed a significant decrease in large fibers (>13 µm in diameter), which are characterized by a lower electrical threshold and a higher conduction velocity (Sima and Robertson, 1978; Norido et al., 1984; Trevillion et al., 2010; Campero et al., 2015). Furthermore, diabetic mice presented a significant increase in small fibers at 32 weeks of age, which could be associated to regenerating axons (Sima and Robertson, 1978; Carson et al., 1980). Whether these structural changes are associated with myelinated or unmyelinated fibers, and whether peripheral fibers of diabetic mice present alterations in myelin thickness remains to be determined. These parameters were previously studied by Nowicki and colleagues (2012) in four-month-old *ob/ob* and *db/db* mice; however, it could be interesting to analyze the evolution of these structural changes during DN progression.

Among other histological parameters analyzed, we found a mild but significant increase in Schwann cell apoptosis. Previously, it has been reported that Schwann cells and neurons are highly susceptible to a diabetic microenvironment (Delaney et al., 2001; Sango et al., 2006; Zhu et al., 2018); however, the nerve physiological consequences resulting from this cell injury are still unclear. Delaney and colleagues (Delaney et al., 2001) demonstrated that hyperglycemia induces Schwann cells apoptosis *in vitro* and *in vivo*, but the proposed cellular mechanisms seem to be multifactorial and include hyperglycemia-dependent injury, endoplasmic reticulum stress, oxidative damage, polyol pathway overactivation, inflammation, growth factors depletion and hypoxia, among others (Delaney et al., 2001; Sango et al., 2006; Dey et al., 2013; Misizin, 2014; Wu et al., 2013; Zhu et al., 2018). The results of our study showed evidence that Schwann cells become gradually more vulnerable to apoptosis as diabetes progresses; however, apoptotic cells were less than 3% of the total analyzed cell population. It remains to be determined whether this small reduction in Schwann cell population could explain some of the functional nerve impairments observed, or whether main Schwann cell defects are not associated with cell death. Other Schwann cell functions that could be affected include secretion of growth factors, myelination, axonal regeneration and impaired metabolism (Mizisin et al., 2014).

We also demonstrated that nerves of diabetic mice displayed a progressive increase of T lymphocyte infiltration. This result has been previously described in diabetic patients' peripheral nerves, and is directly related to the severity of the disease (Cornblath et al., 1990; Younger et al., 1996). Furthermore, lymphocyte infiltration has been described in nerve structures in models of neuropathic pain (Moalem et al., 2004; Cao and DeLeo, 2008); however, the role of immune cells has been poorly explored in diabetic animal models. Thus, it is necessary to dissect the specific role of immune cell contribution to the development of pain, sensitivity changes, inflammation, and a potential role in nerve physiology.

Finally, we explored the relationship between diabetic progression and *vasa nervorum*. Severe abnormalities in the perineurium and the basal lamina surrounding the perineurium in peripheral nerves have been previously described in human patients (Johnson et al., 1981; Misizin, 2014). However, in diabetic animal models the vascular changes seem to be milder than in human patients (Misizin, 2014). Nowicki and colleagues (Nowicki et al., 2012) reported minor changes in microvessels in 4-month-old *ob/ob* and *db/db* mice, but evidenced a significant thickening of the vascular basement membrane. In this work, we did not detect significant changes in nerve vasculature as diabetes progressed. In the same *db/db* animal model, Wang and colleagues (Wang et al., 2015) demonstrated that diabetic mice displayed a decreased local blood flow to the sciatic nerve and a reduction in vessel numbers. However, this analysis was carried out in 44-week-old mice, meanwhile our experimental window does not include mice older than 32 weeks of age. Further experiments are needed to determine whether reduced blood flow affects the trophic support of axon fibers and Schwann cells.

Since an effective DN treatment has not been developed yet, it is necessary to find new therapeutic targets to reverse functional and structural alterations derived from sustained hyperglycemic conditions. In this work, we characterize the progression of main DN features during early-to-advanced phases of the disease in the most used and robust model of T2DM. It is important to note that there is reduced genetic difference in the mouse strain used, generating little variability in the onset and progression of diabetic neuropathy in these mice compared to humans, in which different degrees of functional and structural alterations have been described (Aring et al., 2005).

This study aimed to identify the optimal window for therapeutic interventions. Our results support the general consensus that DN evolves from initial functional to late structural changes, and an effective treatment must be started earlier than the advanced stages of the disease, when functional and histological impairments could be irreversible.

## MATERIALS AND METHODS

### Animals

Spontaneous T2DM transgenic mice line (BKS.Cg-m+/+Lepr^db^/J, stock 000642) was purchased from Jackson Laboratories (Bar Harbor, USA). Female diabetic (*db/db*) and non-diabetic (*db/+*) mice were housed at constant temperature and humidity, with a 12 h light/dark cycle and unrestricted access to standard chow (LabDiet 5P00 RMH 3000, USA) and water. All animal protocols were approved by the Ethics Committee of Facultad de Medicina Clínica Alemana, Universidad del Desarrollo.

### Measurement of blood glucose and biochemical parameters

Non-fasting blood glucose levels were measured using a glucometer (Accu-Chek Performa System, Roche, Basilea, Switzerland). The blood samples were obtained from the tail of alert animals. For biochemical parameters analysis, a group of mice were anesthetized by ketamine/xylazine and blood samples were obtained by cardiac puncture. Glycated hemoglobin (HbA1c) levels were assessed using the DCA2000 analyzer (Bayer Corporation, Leverkusen, Germany). Triglyceride levels were measured using the TG Color GPO/PAP colorimetric kit (Wiener Lab, Rosario, Argentina).

### Quantification of nerve conduction velocity

Motor nerve conduction was determined bilaterally in the sciatic nerves. For this, animals were anesthetized by sevofluorane vapors (Abbot, Tokyo, Japan) and placed on a hot plate to maintain a constant temperature of 32°C. For the determination of motor conduction velocity, the sciatic nerve was stimulated by a supramaximal pulse of 50 µs duration through a commercially available electromyography (Medelec-Teca, Peachtree City, USA) with surface electrodes placed into the sciatic notch and ankle. The distance between the active and referential electrode was kept constant at 6 mm. Signals were amplified with a bandpass 2 Hz–5 KHz analog filter. Signals were analyzed using Medelec software.

### Mechanical withdrawal threshold

Mice were placed in an acrylic box with a mesh floor (Ugo Basile Electronic Von Frey, Varese, Italy) that allowed free access to the plantar surface of the paw. 20 min before recording, mice were allowed to explore the cage in a room with a controlled temperature (25°C). Then, the mid-plantar surface of hind paws were stimulated by the Von Frey filament with an increasing force, and the responses were recorded. The stimulation was repeated three times with an interval of 5 min between stimuli, during three consecutive days. Data were expressed as the mean withdrawal force registered each day.

### Thermal withdrawal latency

Mice were placed in an acrylic box provided with an infrared (IR) light (Ugo Basile Plantar Test, Varese, Italy) 20 min before measuring the withdrawal latency at a room with a controlled temperature (25°C). Then, the IR light (40% of power) was placed beneath of the mid-plantar surface of hind paws, and the withdrawal responses were automatically recorded by the device. We set the cut-off latency at 15 s to avoid hind paw damage. The stimulation was repeated three times with an interval of 5 min between stimuli, during three consecutive days. Data were expressed as the mean withdrawal latency registered each day.

### Intraepidermal nerve fiber density

Mice were euthanized by ketamine/xylazine overdose and cervical dislocation. The foot pads were dissected from the plantar surface of the hind paws, and were fixed in 4% paraformaldehyde (in 0.1 M sodium phosphate buffer) for 15 min at room temperature. The pad samples were immersed in 20% sucrose for 24–72 h at 4°C, and then embedded in Tissue-Tek O.C.T. Compound (Sakura Finetek, Torrance, USA). The pads were cryosectioned at 20 µm, blocked with 5% fish gelatin (with 0.5% Triton X-100 in phosphate buffer) and stained against the axonal protein PGP9.5 (1:150, ab1761 EMD Millipore) and the nuclear staining, DAPI (Applichem, ITW Reagents, Barcelona, Spain). A stack of eight images per foot pad were obtained in a Fluoview FV10i confocal microscope (Olympus, Tokyo, Japan), using 1 µm as optical section thickness. We quantified the number of fibers crossing from dermis to epidermis per linear mm of skin (Christianson et al., 2003).

### T cells infiltration and microvasculature analysis

Sciatic nerves were carefully removed and fixed in 4% paraformaldehyde in phosphate buffer for 24 h at 4°C, and then stored in 30% sucrose for 24–72 h at 4°C. For T cells infiltration analysis, the nerves were embedded in Tissue-Tek O.C.T. Compound, and 10 µm thick nerve cryosections (longitudinally) were mounted on silanized slides. The samples were blocked for one hour in 5% fetal bovine serum, 0.025% Triton X-100, 0.5 M Tris buffer, and incubated overnight (4°C) with anti-mouse CD3 (Dako, Santa Clara, USA, 1:100). The samples were washed three times in 0.025 % Triton X-100, 0.5 M Tris buffer and incubated for 2 h at room temperature with secondary antibody (1:300, anti-rabbit Alexa 555, Cell Signaling Technology, Danvers, USA). The number of CD3^+^ cells was quantified and normalized versus nerve area.

For microvasculature analysis, transversal and longitudinal cryosections (10 µm thick) of sciatic nerves were obtained. The samples were incubated with 1 mg/ml digitonin high purity (Calbiochem-Merk, Darmstadt, Germany) in phosphate buffer, and then were incubated with Isolectin GS-IB4-Alexa 647 (1:100; Life Technologies) overnight. The samples were washed in 1% Triton X-100, 0.5 M Tris buffer and then stained with the nuclear marker Hoescht 33258 (Sigma-Aldrich) in phosphate buffer. The number of Isolectin GS-IB4-alexa 647 positive blood vessels were quantified and normalized versus nerve area. All the samples were analyzed in a Fluoview FV10i confocal microscope.

### TUNEL analysis

TUNEL staining was carried out to detect apoptotic Schwann cells. For this, sciatic nerves were surgically extracted, fixed in 4% paraformaldehyde (24 h at 4°C) and embedded in paraffin. Sections 4 µm thick were mounted in silanized slides (Dako). Apoptotic cells were identified using the In Situ Cell Death Detection kit (Roche), according to the supplier's instructions. Nuclei were counterstained with DAPI (Sigma-Aldrich). We captured the whole nerve in roughly 30 images per sample (>3000 nuclei per nerve) in a Fluoview FV10i confocal microscope. TUNEL positive nuclei were quantified using the cell counter tool (ImageJ software). Data were expressed as the percentage of apoptotic cells.

### Nerve fiber analysis

Sciatic nerves were carefully dissected and fixed in 2.5% glutaraldehyde (Polisciences Inc., Warrington, USA) in a phosphate buffer. Then, the samples were post-fixed in 1% osmium tetroxide and embedded in epoxy resin (Sigma-Aldrich). For nerve fiber analysis, semithin sections (0.5 µm) were obtained, mounted on slides and stained with Toluidine Blue (Sigma-Aldrich). Sections were observed and captured on a light microscope (Axio Imager A1, Zeiss, Oberkochen, Germany), and the area and density of individual fibers were quantified using ImageJ software (NIH).

### Sample size and statistical analysis

All the experiments were performed with an *n*≥6 per experimental condition, unless otherwise specified. Quantitative data were presented as mean±s.e.m. Comparisons between two groups were performed using two-tailed Student's *t*-test, and multiple comparisons were made using one-way ANOVA with Tukey post-test. Grouped, repeated-measures were analyzed with two-way ANOVA with Bonferroni post-test. *P*-values <0.05 were considered statistically significant.

## Supplementary Material

Supplementary information
