# Supplementary material for: Characterization of diabetic neuropathy progression in a mouse model of type 2 diabetes mellitus
Source: Biol Open. 2018 Aug 6;7(9):bio036830. doi: 10.1242/bio.036830 (PMC6176942; doi:10.1242/bio.036830)
Supplement: Supplementary information [file biolopen-7-036830-s1.pdf]

SUPPLEMENTARY FIGURES

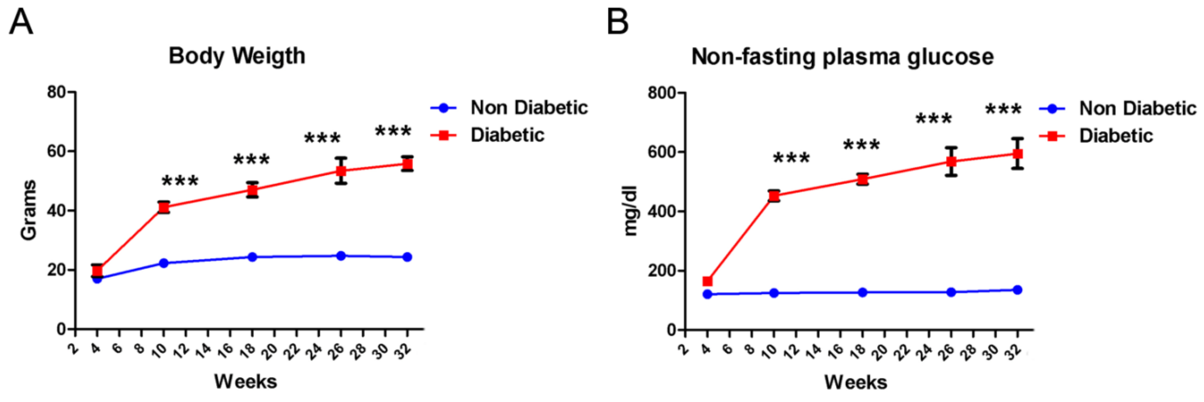

Figure S1. Onset and progression of T2DM in *db/db* mice. (A) Time course of body weight and **(B)** non-fasting plasma glucose levels in diabetic and non-diabetic mice. Data are presented as mean ± S.E.M. Asterisks represent significant differences (two-way ANOVA and Bonferroni post-test, n=10).

Biology Open • Supplementary information

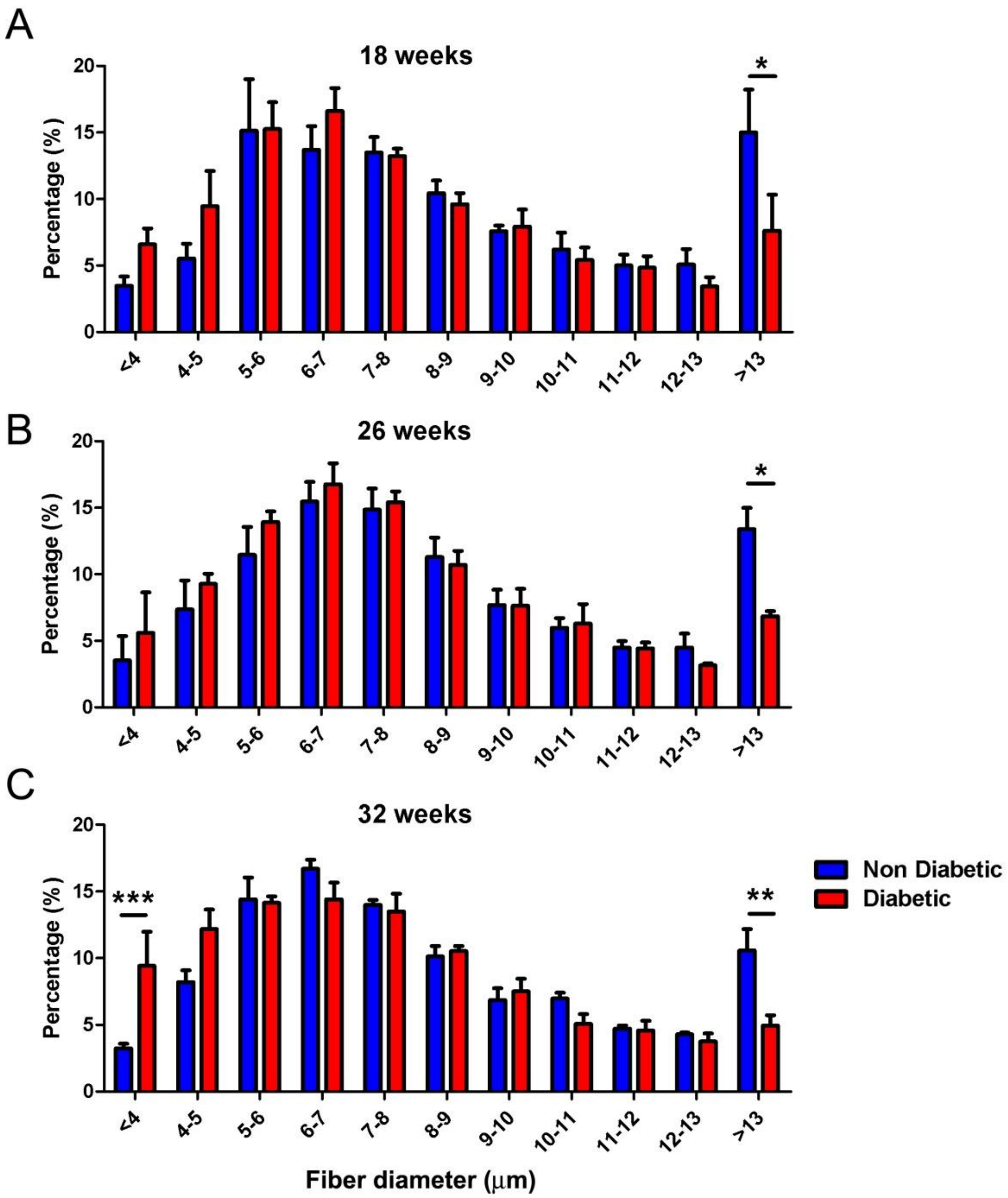

Figure S2. Fiber area distribution changes in sciatic nerves of *db/db* mice. (A-C) Histograms showing the percentage distribution of fiber area in diabetic and non-diabetic mice at 18 (A), 26 (B) and 32 (C) weeks of age. Data are presented as mean ± S.E.M. Asterisks represent significant differences (two tailed Student t-test, n=4).

Biology Open • Supplementary information
